# Multifactorial Design of a Supramolecular Peptide Anti-IL-17 Vaccine Toward the Treatment of Psoriasis

**DOI:** 10.3389/fimmu.2020.01855

**Published:** 2020-08-18

**Authors:** Lucas S. Shores, Sean H. Kelly, Kelly M. Hainline, Jutamas Suwanpradid, Amanda S. MacLeod, Joel H. Collier

**Affiliations:** ^1^Department of Biomedical Engineering, Duke University, Durham, NC, United States; ^2^Department of Dermatology, Duke University School of Medicine, Durham, NC, United States; ^3^Department of Immunology, Duke University School of Medicine, Durham, NC, United States; ^4^Department of Molecular Genetics and Microbiology, Duke University School of Medicine, Durham, NC, United States

**Keywords:** self-assembly, anti-cytokine, active immunotherapy, inflammatory diseases, immunoengineering

## Abstract

Current treatments for chronic immune-mediated diseases such as psoriasis, rheumatoid arthritis, or Crohn's disease commonly rely on cytokine neutralization using monoclonal antibodies; however, such approaches have drawbacks. Frequent repeated dosing can lead to the formation of anti-drug antibodies and patient compliance issues, and it is difficult to identify a single antibody that is broadly efficacious across diverse patient populations. As an alternative to monoclonal antibody therapy, anti-cytokine immunization is a potential means for long-term therapeutic control of chronic inflammatory diseases. Here we report a supramolecular peptide-based approach for raising antibodies against IL-17 and demonstrate its efficacy in a murine model of psoriasis. B-cell epitopes from IL-17 were co-assembled with the universal T-cell epitope PADRE using the Q11 self-assembling peptide nanofiber system. These materials, with or without adjuvants, raised antibody responses against IL-17. Exploiting the modularity of the system, multifactorial experimental designs were used to select formulations maximizing titer and avidity. In a mouse model of psoriasis induced by imiquimod, unadjuvanted nanofibers had therapeutic efficacy, which could be enhanced with alum adjuvant but reversed with CpG adjuvant. Measurements of antibody subclass induced by adjuvanted and unadjuvanted formulations revealed strong correlations between therapeutic efficacy and titers of IgG1 (improved efficacy) or IgG2b (worsened efficacy). These findings have important implications for the development of anti-cytokine active immunotherapies and suggest that immune phenotype is an important metric for eliciting therapeutic anti-cytokine antibody responses.

## Introduction

Anti-cytokine blockade with monoclonal antibodies (mAbs) has revolutionized the treatment of immune-mediated diseases by providing an efficacious treatment modality for multiple inflammatory conditions such as multiple sclerosis, rheumatoid arthritis, and plaque psoriasis (1). Despite incompletely understood etiologies (2), many chronic inflammatory conditions are fueled by a limited set of cytokines which act in concert with immunological and non-immunological cells to induce symptoms of disease (3). In plaque psoriasis, for example, γδ T cells produce the cytokine IL-17, inducing both keratinocyte hyperplasia and the recruitment of neutrophils (4). Inhibition of IL-17 with the monoclonal antibody ixekizumab blocks this recruitment and has been shown to lead to the remission of symptoms in as many as 90% of plaque psoriasis patients receiving injections every 4 weeks. However, the effectiveness of mAbs can both be undercut by secondary resistance due to anti-drug antibodies and difficulties developing mAb subclasses that are most therapeutically effective. To counter these concerns, research has been undertaken to evaluate a new class of cytokine blockade therapies involving immunization against these inflammatory cytokines (5, 6). In some instances, biomaterial platforms are being investigated for this purpose. These therapies could provide an alternative to mAbs and could leverage the ability of biomaterials to induce tailored immune phenotypes in the context of long-term cytokine blockade therapy.

Monoclonal antibodies are manufactured with a uniform specificity (target and affinity for that target) and subclass (function) that dictates the phenotype of the response. While specificity has often been able to be optimized for neutralization, antibody subclass is generally selected for mAb half-life rather than optimal functionality for a specific disease (7, 8). Further, to maintain a therapeutic concentration of mAbs, patients must receive regularly scheduled injections, commonly for the duration of their lives. For example, the dosing of infliximab (anti-TNF) usually occurs once every 8 weeks intravenously, and adalimumab (anti-TNF) and ixekizumab (anti-IL17) are administered once every 2 weeks subcutaneously with adjustments made based on disease severity (9, 10). Anti-drug antibodies that develop against these frequently administered mAbs can lead to secondary resistance to therapy by rapidly clearing mAbs from circulation and can increase risk of adverse effects such as hypersensitivity reactions (11–13). In the case of bococizumab, a mAb designed to reduce LDL-cholesterol by targeting an endogenous kinase, the first long-term study in humans revealed anti-drug antibodies in more than 48% of patients, resulting in reduced outcomes and the cancellation of the drug's development (14).

As an alternative approach that could circumvent these challenges, anti-cytokine immunization has received attention (15). While both anti-cytokine immunization and mAb therapies can target the same inflammatory molecules, anti-cytokine immunization leads to the production of long-lived endogenous polyclonal antibodies and potentially allows for the tailoring of the immune response to elicit antibody subclasses that are most therapeutically effective. Although both approaches target a single epitope of the target cytokine, induced polyclonal antibody responses could have their affinity improved by repeated booster immunizations (16), can avoid any anti-antibody responses from foreign epitopes that vary by population (17), and provides a significantly longer half-life of serum antibody concentration (18, 19). Anti-cytokine immunizations that have been previously investigated have included endogenous cytokines conjugated to protein carriers such as keyhole limpet hemocyanin (KLH) (20) or virus-like particles (VLP) (21–23). In both cases, the carriers supply exogenous T cell epitopes to enable B cells to produce anti-cytokine antibodies in a T-dependent fashion (22, 24). Anti-IL-17A immunizations have previously been shown to induce antibodies when whole IL-17A was conjugated to ovalbumin protein (25), when conjugated to VLPs (26, 27), or when an IL-17 peptide epitope was conjugated to VLPs (28). A few of these approaches have reached clinical trials for various cytokines, but with limited success in terms of therapeutic efficacy (29–32).

While carrier proteins have offered one route for anti-cytokine immunotherapies, materials research has begun to redefine vaccine development by emphasizing how physical parameters such as size (33, 34), shape (35, 36), charge (37), and other materials properties can alter the immune response. Recently, our group has pursued anti-cytokine immunization via a supramolecular nanofiber platform based on the self-assembling peptide Q11 (Ac-QQKFQFQFEQQ-NH_2_) (38). In contrast to carrier proteins, the use of supramolecular nanofibers facilitates the straightforward assembly of different immunological epitopes together in finely controlled ratios (37, 39–42). This modularity enables the application of multifactorial Design of Experiments (DoE) methodologies to investigate and optimize various co-assembled epitopes (43). In this context, previous work has shown that changing the T cell epitope content can alter both the T cell and B cell response of an anti-cytokine immunization (42). In an active immunotherapy raising antibodies against TNF, it was shown that prior immunization with Q11-based nanofibers bearing TNF B-cell epitopes and exogenous T-cell epitopes could partially protect mice in a model of acute inflammation (38).

Here we have expanded upon these previous observations to design peptide nanofibers raising antibodies against IL-17, a central cytokine in the pathophysiology of multiple diseases, particularly psoriasis (44–46). We investigated two anti-IL-17 peptide epitopes and demonstrated their ability to assemble into nanofibers and induce antibodies in an adjuvant-free context. Using a DoE approach, we elucidated that the B and T cell epitope density, as well as the ratio between these two components, strongly influenced the strength and quality of the anti-IL-17 antibody response. Finally, we evaluated optimized formulations in a murine model of psoriasis, finding that therapeutic efficacy is strongly correlated with the subclass of IgG antibodies raised.

## Methods

### Epitope Selection

B-cell epitopes for investigation were selected using the Kolaskar Tongaonkar Antigenicity Test, which uses known antigenic sequences and amino acid neighbors to produce predictions with reported 75% accuracy (47). Scores from the murine sequence of IL-17 were then compared to known crystal structures from its human homolog. One previously reported epitope (28) also returned high scores using this tool and was evaluated for antigenicity within the Q11 platform. Two epitopes identified for this study were named IL17.1 and IL17.2 to avoid confusion with alternative IL17 isoforms (IL17A and IL17B). All peptide sequences studied are listed in Supplementary Table 1.

### Solid Phase Peptide Synthesis

Peptides were synthesized as previously reported (48). Briefly, peptides were synthesized using microwave-assisted solid phase peptide synthesis on a Liberty Blue Peptide Synthesizer (CEM) with standard FMOC chemistry. Peptides were cleaved from the resin with trifluoroacetic acid and precipitated in diethyl ether prior to purification using reverse phase HPLC. Peptide identity was confirmed using MALDI mass spectrometry, and purity was assessed to be >80% by analytical HPLC.

### Preparation of Immunization Formulations

Nanofibers were formed by mixing dry purified peptide for 10 min prior to adding sterile water to form an 8 mM peptide solution. This solution was incubated overnight at 4°C prior to adding 10x PBS (Corning 46-013-CM) and additional sterile water to form a 2 mM peptide solution in 1x PBS, which was incubated at room temperature for 3 h before being used for further studies. For immunizations containing CpG (ODN 1826, IAX-200-002-3001 Innaxon), CpG was added to peptide solutions at a concentration of 100 μg/mL, after overnight fibrillization (at the same time as the addition of 10x PBS), while for immunizations containing Alum (Invivogen Alhydrogel 2%), the adjuvant was mixed at a 1:1 concentration with previously formed nanofibers for 10 min.

### Transmission Electron Microscopy

Peptide nanostructure was evaluated using Transmission Electron Microscopy (TEM) on an FEI Tecnai G^2^ Twin. Peptides were fibrillized for 3 h, diluted to 0.2 mM in PBS as previously reported (37), applied to formvar copper grids (Electron Microscopy Sciences, EMS400-Cu), and negative-stained with uranyl acetate 1% (w/v) in distilled water (Electron Microscopy Sciences).

### Thioflavin T Binding Assay

As a measure of fibrillization, the Thioflavin T (ThT) assay was utilized. For these measurements, 20 μL of 2 mM peptide solutions were mixed with 180 μL of a 50 μM solution of ThT (Alfa Aesar, J61043) in 1X PBS in a black 96-well plate and read using a Molecular Devices Spectramax M2 spectrophotometer (excitation at 440 nm, emission at 488 nm).

### Immunizations

Female, wild-type C57BL/6 mice (Envigo) 8–12 weeks of age at the start of experimental protocols were used for all animal studies. Animal experiments were approved by the Institutional Animal Care and Use Committee of Duke University in compliance with the NIH Guide for the Care and Use of Laboratory Animals. Mice were anesthetized with isoflurane prior to subcutaneous immunization. For prime and boost immunizations, mice were injected subcutaneously with 50 μl of 2 mM peptide nanofiber solutions in PBS on their left and right shoulder. In all studies, mice were boosted with the same dose at week 2.5 and 5 after primary immunizations, with or without adjuvants indicated. For CpG, 10 μg per mouse per immunization was administered according to manufacturer guidelines. For alum, 500 μg per mouse per immunization of alum was administered according to manufacturer guidelines. Blood was collected for analysis at weeks 2, 4, and 7 unless otherwise indicated.

### Enzyme-Linked Immunosorbent Assay (ELISA)

ELISA was conducted as previously described (38, 48). For total IgG ELISA, plates were coated with streptavidin overnight, and then either coated with biotinylated peptide solution (20 μg /ml) or PBS. A reported titer of 1 indicates no detectable signal above background. For measuring antibody responses against whole protein, murine IL-17A (R&D Systems #421-ML-025) was coated at 5 μg/mL overnight in sterile 4 mM HCl containing 0.1% w/v bovine serum albumin. Background wells were coated with the same solution lacking IL-17A. Plates were then blocked with a 2% (w/v) BSA solution. Serum was diluted and coated onto plates for 2 h prior to incubating with an antibody targeting the conserved Igκ fragment (Jackson ImmunoResearch #115-035-008). For the antibody avidity rank assay, 50 μl of 5M urea (Sigma) was added to half the wells and incubated for 5 min before washing and adding detection antibody. For antibody isotyping, isotype specific-antibodies (Southern Biotech #1010-05) were used in place of the total IgG detection antibody while all other steps were similar.

### Enzyme-Linked Immune Absorbent Spot (ELISpot) Assay

T cell responses to immunizing peptides were measured by ELISpot assay. Mice were sacrificed 7 days after a booster vaccination to measure T cell responses (58 weeks after the initial immunization). Splenocytes purified from mice were plated at a density of 250,000 spots per well of a 96-well ELISpot plate (Millipore, MAIPSWU10) and stimulated with 5 μM soluble IL17.1 or PADRE peptide, left untreated, or stimulated with Concanavalin A (Con A) (Sigma, C5275) which non-specifically activates T cells. Biotinylated capture-detection antibody pairs for either IL-4 (BD Cat. 551878) or IFNγ (BD Cat. 551881) were used according to manufacturer's guidelines in conjunction with streptavidin-alkaline phosphatase (Mabtech, 3310-0) and Sigmafast BCIP/NBT (Sigma, B5655). After development, plates were evaluated for spot count by Zellnet Consulting using a Zeiss KS ELISpot reader.

### Imiquimod-Induced Psoriasis

To induce psoriasis, hair was removed from the backs of mice by plucking, and 0.125 g 5% imiquimod cream [6.25 mg imiquimod (Perrigo)] was applied topically daily for 5 consecutive days. Twenty four hours after the last application, mice were sacrificed and skin was harvested for histology. Mice were immunized prophylactically at 8 weeks of age, with a boost 2.5 weeks afterwards, and imiquimod treatment was initiated 5 weeks after the primary immunization. Only one boost was administered to avoid the complication of barbering, a well-documented, age-dependent phenomenon in C57BL/6 mice (49) that interfered with the onset of IMQ-induced psoriasis in our hands. Anti IL-17 antibody was used as a control (Bio-X-Cell clone 17F3, #BE0173, 250 μg administered IP at 3 days before and again on the same day as the initial imiquimod application). Body weight and spleen weight were recorded at the endpoint (immediately after euthanization).

### Histology

Skin was harvested from the backs of mice and submerged in OCT solution prior to freezing with dry ice. Sections 18 μm thick were then collected (Thermo Scientific CryoStar NX50), stained with Toluidine Blue O (Harleco 364-12), and imaged with brightfield microscopy (EVOS FL Auto). Measurements of epidermal thickness in interfollicular regions were taken with ImageJ software at 4 locations *per section* from two different sections by an individual blinded to the experimental conditions. Follicles were not included in these measurements.

### Statistical Analysis

For statistical analyses using multifactorial designs, JMP14 Pro software was used to determine statistical significance and power calculations. In multifactorial experiments, tests for main effect and interaction parameter were carried out using generalized linear regression models. For traditional one-factor at a time analyses involving one-way ANOVA, multiple comparisons, and individual *t*-tests, Graphpad prism software was used. Specific analyses are described in the figure legends.

## Results

### Epitope Selection and Nanofiber Characterization

**Figure 1:**
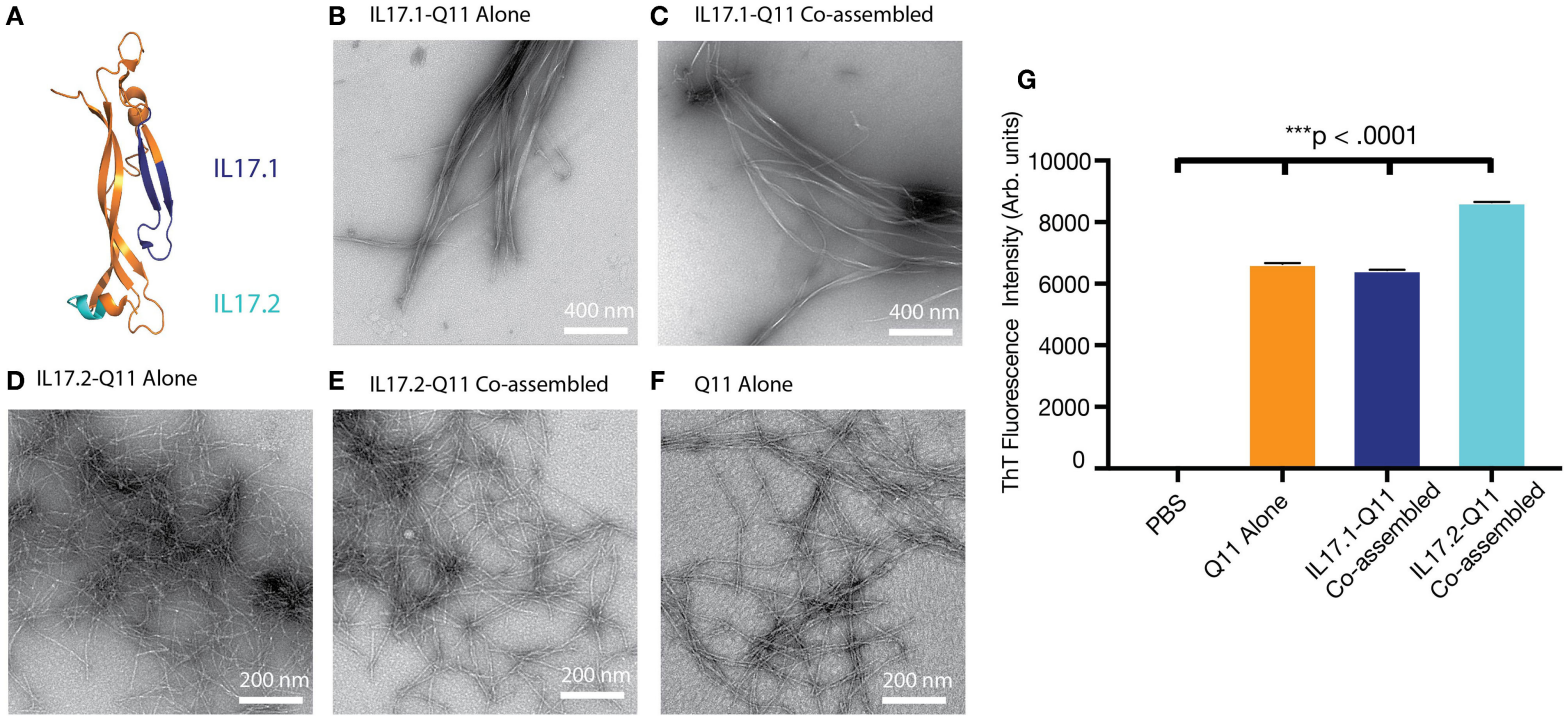
Selection of IL-17 epitopes and characterization of IL17.1 and IL17.2 nanofibers. Two peptide epitopes, IL17.1 and IL17.2, were identified within mouse IL17A. **(A)** Homologous epitopes are shown within the crystal structure of human IL17A. **(B–F)** Both IL17.1-Q11 and IL17.2-Q11 peptides self-assembled into nanofibers (imaged using negative-stained TEM) alone or when co-assembled with Q11 and PADRE-Q11 (single peptides formulated at 2 mM; co-assembled peptides formulated at 1 mM IL17.1-Q11 or IL17.2-Q11/0.95 mM Q11/0.05 mM PADRE-Q11; all nanofibers diluted 10-fold before application to TEM grids). **(G)** All formulations had significant β-sheet character by ThT fluorescence (mean ± SD, *n* = 3, all groups significantly different from all other groups, ***p < 0.001 by one-way ANOVA and Tukey's Multiple Comparisons test).

In order to develop an active immunotherapy capable of raising antibodies against a focused B cell epitope within IL-17A, we utilized Kolaskar Tongaonkar antigenicity predictions (47) and surveyed previously published B-cell epitopes (Figure S1). Two potential B cell epitopes were selected: IL17.1 was selected from a previous report employing virus-like particles (28) and IL17.2 was selected from antigenicity prediction and its apparent availability on the protein surface (Figure 1A). These epitopes were then synthesized in tandem with the Q11 self-assembling sequence.

Both IL17.1-Q11 and IL17.2-Q11 were found to assemble into nanofibers by TEM alone (Figures 1B,D) or when co-assembled with Q11 and PADRE-Q11 in a 1.0:0.05:0.95 molar ratio (Figures 1C,E). Interestingly, IL17.1-Q11 formed fibers that appeared more tape-like than Q11 nanofibers (Figure 1A), while IL17.2-Q11 formed assemblies more similar to Q11. Despite minor morphological dissimilarities, ThT binding indicated that both IL17.1-Q11 and IL17.2-Q11 formed structures with significant β-sheet character and maintained this character when co-assembled with Q11 (Figure 1G, Figure S2). The IL17.1 peptide lacking the Q11 assembly domain did not exhibit ThT fluorescence significantly above that of PBS (Figure S2), whereas, the soluble IL17.2 peptide did exhibit ThT fluorescence, potentially indicating that it had some capacity to assemble even without conjugation to Q11, yet this aspect was not explored further. In sum, owing to their capacity to form nanofibers both alone and when mixed with Q11 and PADRE-Q11, both IL17.1-Q11 and IL17.2-Q11 were carried forward into evaluations of immunogenicity.

### IL-17 B-Cell Epitope Evaluation

**Figure 2:**
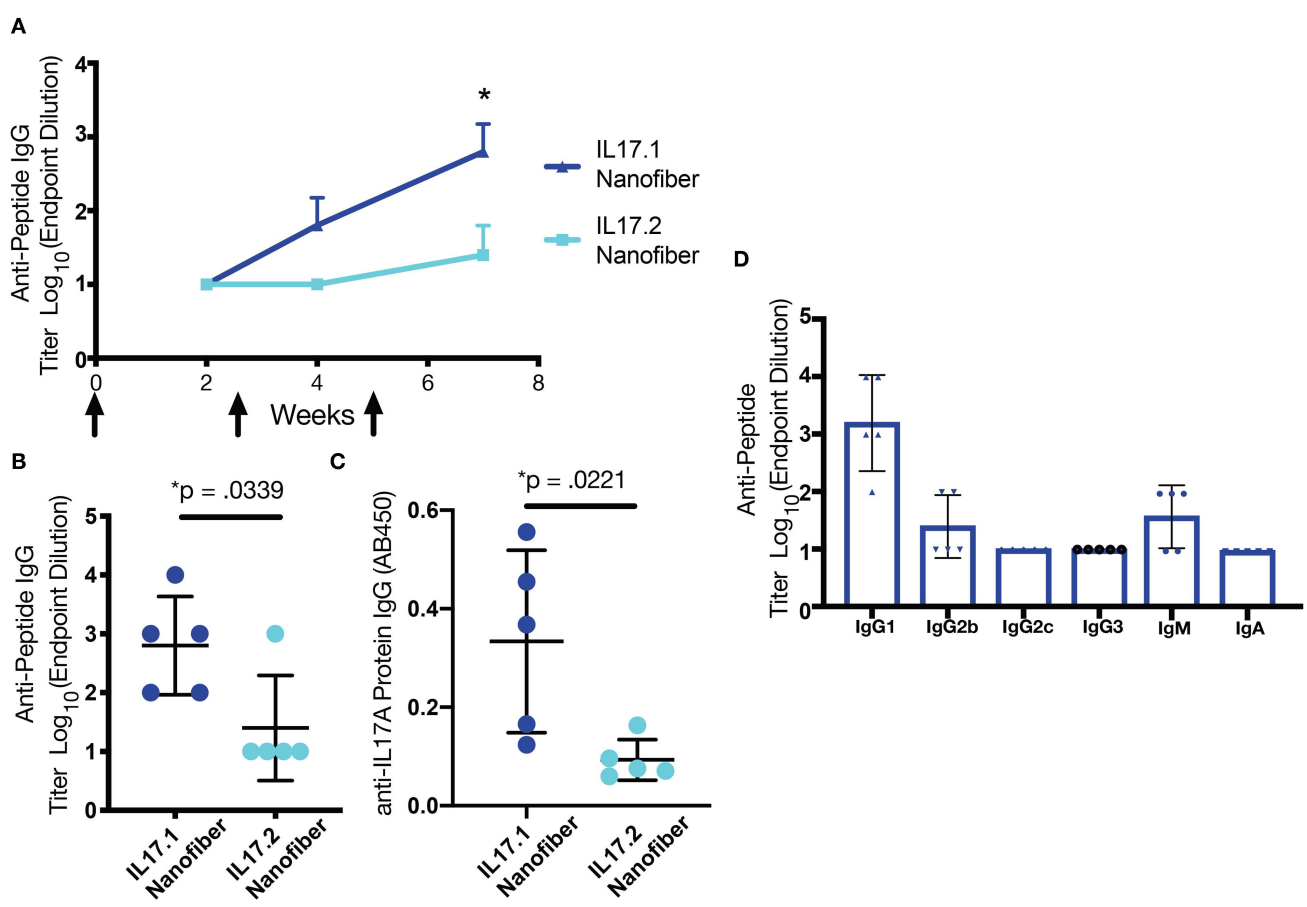
B cell epitope screening in mice. **(A)** IL-17.1-Q11 and IL17.2-Q11 peptides were co-assembled into nanofibers with Q11 and PADRE-Q11 and injected subcutaneously without adjuvant at weeks 0, 2.5, and 5. Antibody titers were measured at weeks 2, 4, and 7. Mice immunized with IL17.1 raised significantly higher antibody titers against both immunizing peptide **(B)** and IL17A protein **(C)** compared to mice immunized with IL17.2. Analysis of the antibody isotype raised by IL17.1 demonstrated a bias toward IgG1 **(D)**. Statistical comparisons were conducted by Student's *t*-test (*n* = 5), *p < 0.05. Data are presented as mean ± SD.

Self-assembled formulations were subsequently tested for immunogenicity in mice. Mice were immunized with nanofibers containing IL17.1-Q11 or IL17.2-Q11 co-assembled with Q11 and PADRE-Q11, to provide nanofibers presenting both B cell epitopes from IL-17 and the universal T-helper epitope PADRE (50, 51). Mice were boosted at weeks 2.5 and 5, and antibody responses were measured at weeks 2, 4, and 7. For nanofibers containing IL17.1-Q11, all mice produced antibodies targeting the B-cell epitope peptide (Figure 2A, Figure S3). In contrast, only one of five mice produced antibodies against IL17.2-Q11 (Figure 2B). Further, when serum was tested against recombinant murine IL-17A, IL17.1 immunization produced a significantly higher response against the protein (Figure 2C). IL17.1 was accordingly chosen to advance into further study owing to its superior immunogenicity. Further characterization of the IL17.1 antibody response revealed that there was predominantly an IgG1-focused response with only 2 mice producing detectable titers of IgG2b at the lowest dilution tested. This potentially indicated a predominantly Th2 response, which corresponded with previous observations of Q11-based immunizations also raising predominant Th2 responses (38).

### Q11-Based Vaccination Produced a Long-Lived Response Against IL17.1

**Figure 3:**
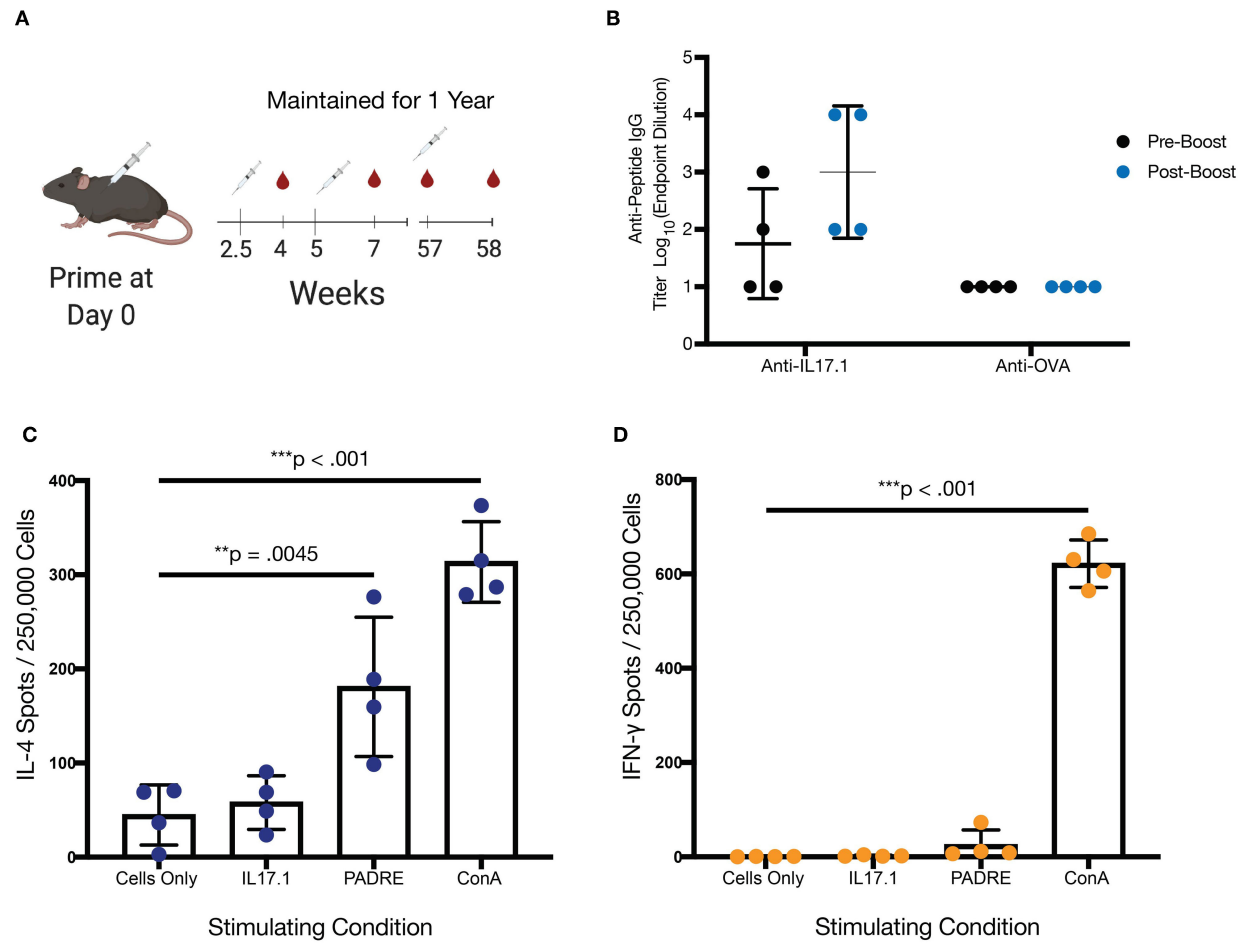
Long-term immunization with IL-17.1 maintained immune memory without an autologous anti-IL17.1 T-cell response. **(A)** Mice were maintained for 1 year after the final immunization before blood collection and a subsequent boost, with the exception of one mouse that died of unrelated causes at 50 weeks. Final sera were collected at week 58 and mice were sacrificed for analysis of T cell response by ELISpot. **(B)** Anti-IL17.1 responses had declined by 1 year but rebounded after being recalled (unrelated antigen (OVA) responses included as a negative control). The difference was measurable although not statistically significantly different (ns, *p* = 0.1411) **(C)** Splenocytes were left unstimulated or stimulated with IL17.1 peptide, PADRE peptide, or Concanavalin A and demonstrated a significant IL-4 response against PADRE but no significant response against IL17.1. **(D)** Neither IL17.1 nor PADRE elicited a significant IFN-γ response. *n* = 4, statistical differences for antibody responses were calculated by paired *t*-test, while statistical differences for the ELISpot assay were calculated by one-way ANOVA and Tukey's test for multiple comparisons. **p < 0.01, ***p < 0.001. Data are presented as Mean ± SD.

Mice from the original IL17.1 trial were maintained for 1 year after the previous immunization schedule (Figure 3A). While as a group, mice at 57 weeks after immunization did not maintain statistically significant titers compared to unvaccinated mice, two of four mice maintained measurable titers, indicating that long-lived responses are possible. Moreover, the group responded to boosting with a recall response, with measurable, antigen-specific titers after 1 week in all four mice. This response did not cross-react with other peptide epitopes such as the OVA epitope from the model antigen ovalbumin (Figure 3B, Figures S4, S5). These kinetics are faster than observed for primary immunizations (Figure 2), suggesting a recall response. Though antibody titer measurement at week 58 was not significantly higher than the pre-boost (ns, *p* = 0.1411) it was significantly higher than antibody response at 2 weeks (^**^, *p* = 0.0056). Following this boost, spleens were harvested from mice at week 58, and the T cell responses to the IL17.1 peptide and the PADRE T cell epitope were analyzed. Strikingly, IL17.1 produced no detectable T cell responses, while PADRE stimulation indicated a significant population of responding T cells producing IL-4 but not IFN-γ (Figures 3C,D). This indicated a T cell response that was PADRE-specific but not IL17.1-specific, an important consideration for anti-cytokine immunization which aims to avoid breaking T cell tolerance (24).

### Multifactorial Optimization

**Figure 4:**
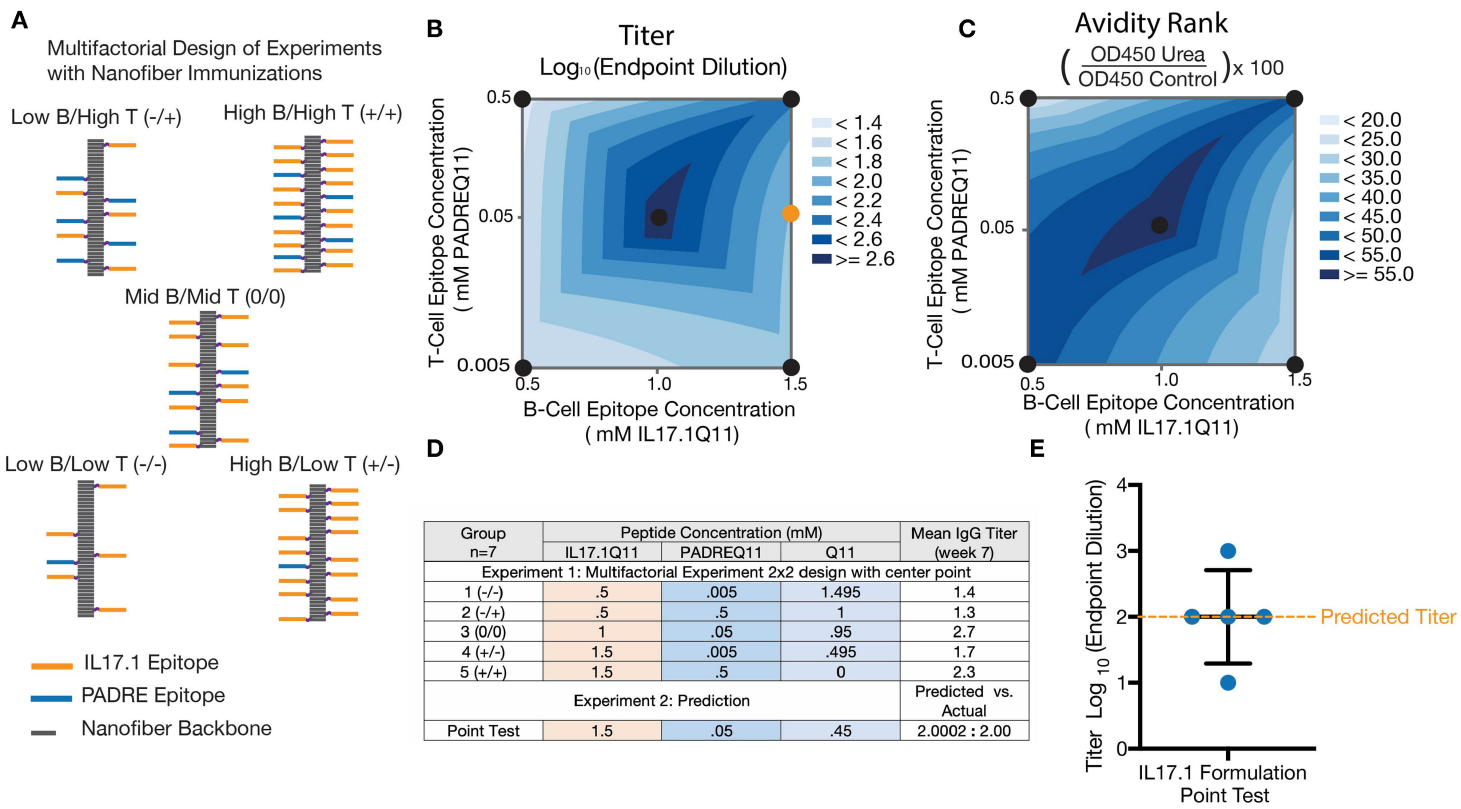
Multifactorial optimization of nanofibers containing IL17.1 B-cell epitopes and PADRE T-cell epitopes. Design of Experiments (DoE) methodology **(A,D)** was employed to investigate how T-cell epitope and B-cell epitope content influenced anti-IL17 titers **(B)** and avidity **(C)**. A 2 × 2 multifactorial design with a center point was utilized, using nanofibers with stoichiometrically controlled amounts of B- and T-cell epitope indicated (*n* = 7). To test the predictive power of the DoE, a formulation not specifically tested in the original design was tested subsequently (Formulation shown by the orange dot in B). **(E)** Average titers generated by this formulation corresponded closely with the DoE prediction (*n* = 5, individual mice shown, with mean ± SD indicated).

Multifactorial experimental designs are commonly employed in a broad range of technical fields to efficiently optimize a desired outcome while simultaneously gaining knowledge about how multiple factors may interact. Although traditionally underutilized in biological settings, such designs have been receiving increasing attention recently (43, 52–54). We chose to employ this methodology to investigate how epitope content influenced overall titer and avidity, using a 2 × 2 full factorial design with a center point (Figures 4A,D). Based on previous research analyzing the impact of altering T cell epitope content alone (42), we chose three concentrations of T cell epitopes at different orders of magnitude and B cell epitopes over a linear range. All nanofibers were formed using a total peptide concentration of 2 mM, using unmodified Q11 peptide as a base material (Figure 4A). Based on previously published data comparing anti-cytokine responses at different T cell epitope ratios (38), we used JMP14 to select a sample size of 7 mice per group to provide an estimated 80% power for this experiment.

Analysis of these results by two-way ANOVA indicated that the B cell epitope content in nanofibers had a significant main effect influencing antibody titer (*p* = 0.0242) within the range tested. The T cell epitope content (*p* = 0.4305) and the interaction parameter (*p* = 0.1940). having a non-significant effect (Figure 4B). Antibody titer is a combined metric which is dependent on both antibody concentration in the serum and antibody avidity for the target. To better assess the antibody avidity, a urea assay was used on serum from the same 35 mice. Interestingly, avidity exhibited distinct differences compared with the response shown for antibody titers, with no significant main effect of either epitope density (B cell: *p* = 0.9808 and T cell: *p* = 0.9188) but a strongly significant effect for the interaction parameter (*p* = 0.0054) (Figure 4C). This suggested that neither the T cell nor B cell epitope alone were responsible for the changes in avidity noticed here, but rather the ratio of the T and B cell epitope density.

The predictive value of the model generated was tested by evaluating a new formulation not included in the original experimental design. A group of 5 mice was immunized with nanofibers containing a previously untested epitope ratio (shown schematically as an orange circle in Figure 4B, titer data shown in Figure 4E), and antibody titers were compared to the value predicted by the factorial design. The mean titer measured in this experiment closely matched the predicted antibody titer at week 7, supporting the validity of this model to predict responses not included in the original design. Fortuitously, the center of the design was selected as the formulation to advance into further studies with adjuvants and in a mouse model of psoriasis. All further experiments were conducted with this optimized nanofiber immunization formulation.

### Adjuvants Enhanced Antibody Responses and Influenced Antibody Subclass

**Figure 5:**
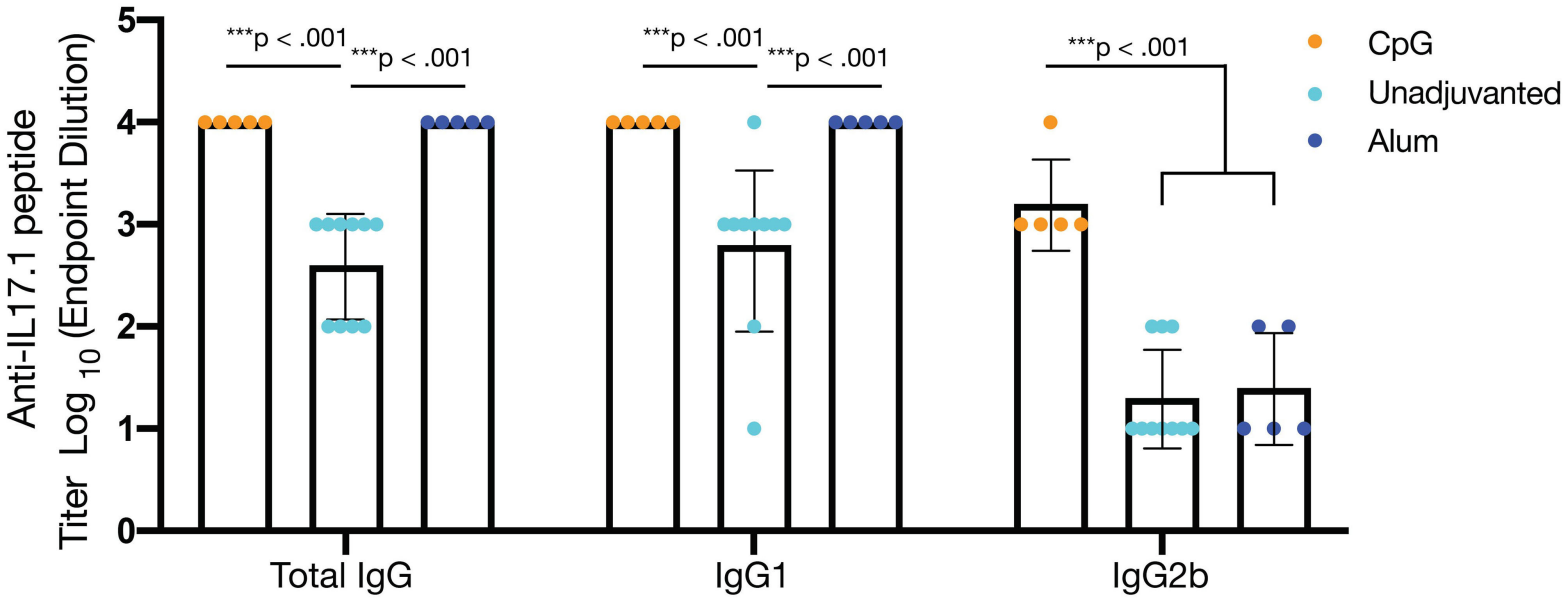
Adjuvants impact antibody titer and alter IgG subclass. To enhance antibody titers, optimized nanofibers containing IL17.1 B cell epitopes and PADRE T cell epitopes were administered along with the adjuvants shown. CpG (*n* = 5) and alum (*n* = 5) enhanced total IgG and IgG1 anti-IL17.1 IgG titers compared to unadjuvanted controls (*n* = 10), but only CpG adjuvant significantly enhanced anti-IL17.1 IgG2b titers, indicating a more Th1-like phenotype. Data shown represent two separate experiments: Experiment 1: CpG *n* = 5 and unadjuvanted *n* = 5, Experiment 2: Alum *n* = 5 and unadjuvanted *n* = 5. ***p < 0.001 by one-way ANOVA and Tukey's test for multiple comparisons. Data are presented as means ± SD, with individual mice indicated.

Although it is not yet clear what titers of anti-IL-17 antibodies will be most protective in the context of various inflammatory conditions, the antibody titers measured in the study thus far against optimized IL17.1-containing nanofibers were considered to be only moderate. To investigate the extent to which antibody titers could be augmented, we investigated formulations containing the adjuvants CpG (ODN 1826) or alum (Alhydrogel, Invivogen). Both adjuvants were able to significantly increase (*p* < 0.001) the anti-IL17.1 IgG titer compared to the unadjuvanted formulation, but the two adjuvants also had distinct effects on IgG antibody subclasses elicited (Figure 5, Figure S6). Alum produced stronger total IgG and IgG1 responses but did not influence IgG2b, whereas CpG produced stronger IgG2b responses. These results were expected, as they corresponded with previous observations that alum-based adjuvants tend to skew the immune response toward an IgG1-dominated Th2 phenotype, while CpG skews toward an increased IgG2b phenotype associated with Th1 responses (55). These differences in IgG antibody subclass have been shown previously to influence cellular engagement with antibody-bound antigens. For example, mouse IgG1 interacts with activating receptor FcγRIII and inhibiting receptor FcγRIIB, which are found on all myeloid cells, while mouse IgG2b additionally binds the activating receptors FcγRI on monocyte-derived dendritic cells and FcγRIV on Ly6clo monocytes, macrophages, and neutrophils (56). While beneficial for the clearance of viral infections, the additional cellular recruitment and activation from IgG2b has not been thoroughly investigated for its role in anti-cytokine immunizations to our knowledge.

### Reduction of Epidermal Thickening in a Model of Psoriasis

**Figure 6:**
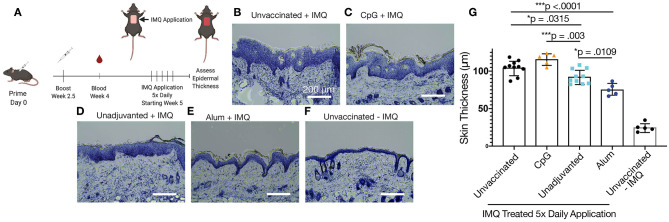
IL17.1 nanofiber immunizations diminished imiquimod-induced psoriasis. Mice were immunized prophylactically at 8 weeks of age and boosted 2.5 weeks afterwards. Five weeks after primary immunization, imiquimod (IMQ) was applied daily for 5 days, and tissues were harvested on the sixth day **(A)**. Groups included unvaccinated mice **(B)**, mice vaccinated with CpG-adjuvanted nanofibers **(C)**, unadjuvanted nanofibers **(D)**, and alum-adjuvanted nanofibers **(E)**, plus mice that were neither vaccinated nor administered imiquimod **(F)**. Epidermal thickness was compared at 8 locations over two sections from each animal **(G)**. Statistical significance was measured by one-way ANOVA and Tukey's Multiple Comparisons *post-hoc* analysis. *p < 0.05, ***p < 0.001. Data are presented as means ± SD. Data shown represent two separately conducted experiments: *Experiment 1:* unvaccinated + IMQ *n* = 5, CpG-adjuvanted nanofibers *n* = 5, unadjuvanted nanofibers *n* = 5, unvaccinated without IMQ *n* = 5; *Experiment 2:* unvaccinated + IMQ *n* = 5, alum-adjuvanted nanofibers *n* = 5, unadjuvanted nanofibers *n* = 5. See Figure S7 for statistical justification for combining these experiments in the analysis shown in **(G)**.

Mice immunized with variously adjuvanted nanofibers were then studied with a model of psoriasis in which the application of imiquimod leads to epidermal thickening (Figure 6A) mimicking plaque psoriasis in humans in addition to the increase of IL-17 and other inflammatory cytokines. Epidermal thickness, which correlates with the expression of these cytokines, was chosen for the sole metric of outcome. In this model, mice that had been previously immunized with unadjuvanted nanofibers showed an improvement compared to unvaccinated controls, exhibiting reduced epidermal thickening (Figures 6B,D, *p* = 0.0315). An even greater therapeutic effect was observed for alum-adjuvanted nanofibers, which significantly reduced epidermal thickening compared to unvaccinated (*p* < 0.0001), CpG-adjuvanted (*p* < 0.0001), and unadjuvanted (*p* = 0.0109) nanofiber formulations (Figures 6E,G). Strikingly, despite elevated IgG1 and total IgG titers compared to unadjuvanted controls, CpG-adjuvanted nanofibers exhibited the highest average epidermal thickness among all groups tested (Figures 6C,G), suggesting an exacerbation of psoriatic symptoms compared to unimmunized or immunized groups, although comparisons to unimmunized mice did not reach statistical significance. All imiquimod treated groups exhibited increased epidermal thickening compared to control mice that were not administered imiquimod (Figure 6F). A commercially available IL17 mAb was then compared to these samples in age-matched mice, with no significant differences observed between nanofiber immunization and anti-IL17 mAb treatment (Figure S8).

**Figure 7:**
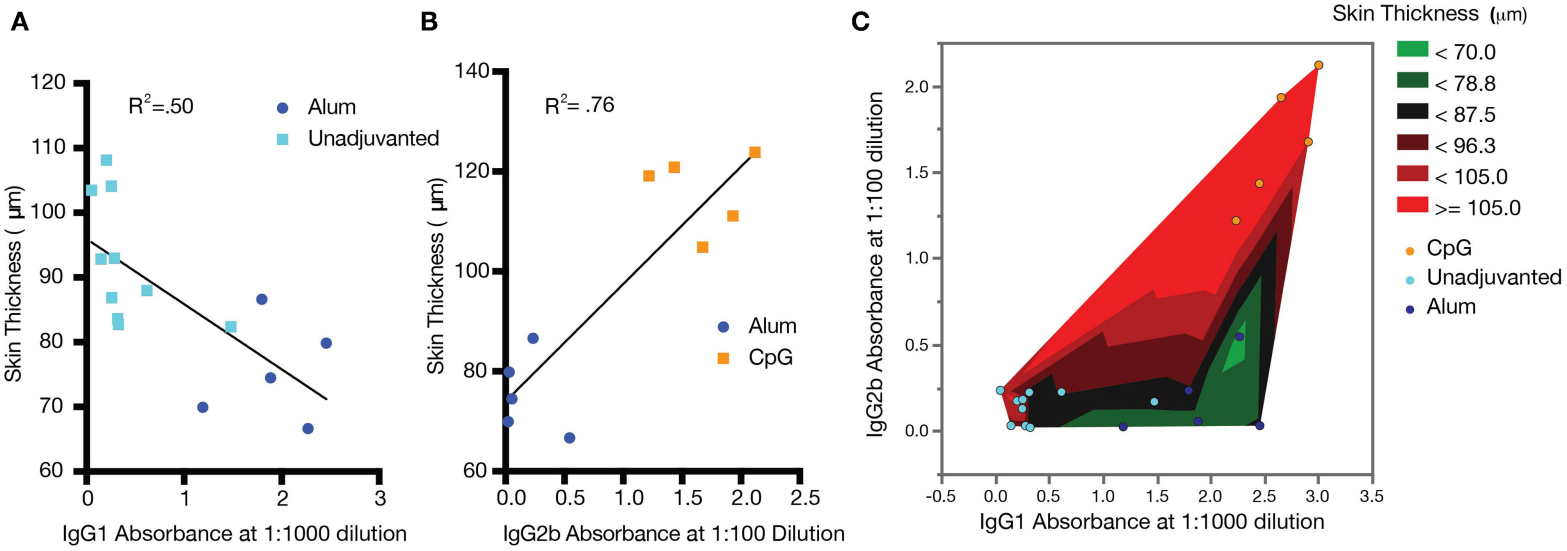
Correlations between antibody titer, subclass, and psoriasis severity. Increased IgG1 correlates with improved psoriasis (decreased epidermal thickness) **(A)**, while increased IgG2b correlates with exacerbated psoriasis (increased epidermal thickness) **(B)**. A heatmap of epidermal thickness illustrates a target region (green) for reduced epidermal thickening **(C)**. Analysis was carried out by simple linear regression. Data shown represent a combination of two separately conducted experiments: Experiment 1: CpG *n* = 5 and unadjuvanted *n* = 5; Experiment 2: alum *n* = 5 and unadjuvanted *n* = 5.

While the role of antibody subclass has been investigated thoroughly in the context of infectious disease, comparatively little work has been done to clarify the effects of subclass in anti-cytokine immunization. To further analyze the relationship between epidermal thickening and antibody subclass in this model, the absorbance of IgG2b at a dilution of 1:100 and IgG1 at a dilution of 1:1000 was compared. For unadjuvanted vs. alum-adjuvanted responses, where IgG2b titer was constant, IgG1 absorbance negatively correlated with epidermal thickening (Figure 7A, *R*^2^ = 0.50, *p* < 0.01). That is, increased IgG1 was associated with reduction in the severity of psoriasis symptoms. In contrast, for alum- and CpG-adjuvanted groups where IgG1 titer was constant, IgG2b absorbance *positively* correlated with epidermal thickening (Figure 7B, *R*^2^ = 0.76, *p* < 0.01). That is, IgG2b was associated with a *poorer* ability to reduce psoriasis symptoms and potentially an exacerbation of symptoms. Plotting this data as a heatmap of epidermal thickening, the optimal condition of low IgG2b and high IgG1 response becomes clearly visible (Figure 7C). Of course, these data are correlative at present and are shown to suggest future mechanistic and immunologically controlled experiments clarifying causal relationships between IgG subclass and therapeutic efficacy of anti-cytokine vaccines.

## Discussion

Peptide nanofiber immunization against autologous targets offers a means to control both the magnitude and phenotype of therapeutic antibody responses for the long-term treatment of inflammatory diseases. While antibody responses have primarily been controlled by changing innate cell signaling, we demonstrate here how B- and T-cell epitope content can influence responses in a supramolecular peptide-based vaccine, utilizing DoE methodologies to demonstrate how both titer and avidity can be influenced by these inputs. We then demonstrate how phenotypes of the immune response can have a significant impact on the course of imiquimod-induced psoriasis. These results demonstrate how a peptide-based approach can allow for highly specific targeting of cytokines—both capitalizing on the strengths and buttressing the weaknesses of monoclonal antibody-based therapeutics.

Peptide immunization for anti-cytokine vaccination offers an opportunity to increase specificity of antibody responses, avoiding off-target effects which have hindered other anti-cytokine efforts such as non-neutralizing immunodominant epitopes and unwanted reactions against epitopes shared between cytokines (6). However, the reduced immunogenicity of peptides has historically posed a challenge (57). Soluble peptides rarely elicit high-titer, long-lasting antibody responses without adjuvants, but biomaterials such as supramolecular nanofibers can be employed to produce long-lived, high-titer responses from these otherwise non-immunogenic peptides, in many cases without adjuvants (37, 40, 58–60). These nanofiber materials have also been demonstrated to be stable, maintaining immunogenicity *in vitro* under prolonged conditions which otherwise cause loss of immunogenicity for complex folded proteins (61). Nanofiber vaccine responses can be controlled by B-cell and T-cell epitope content, representing a modulation of adaptive immune signals as opposed to the more common approach of modulating antigen presenting cell signaling to change co-stimulation and cytokine production (62–65). Further, biomaterials with high modularity, such as supramolecular assemblies, are particularly amenable to engineering strategies such as Design of Experiments methodologies. These statistical constructs can be employed for optimization and can also provide insight into interactions between factors in a way that is more informative than simple presence/absence or one-factor-at-a-time experiments.

In addition to advantages offered by the modularity of Q11 with respect to changing epitope content, these materials can also be formulated with adjuvants to further shape the phenotypes of antibody responses (59). By themselves, Q11-based vaccines have been shown repeatedly to not induce pro-inflammatory cytokines, representing a relative “blank slate” that can be combined with additional adjuvants or immune modulators to further shape or augment responses (41, 58). In contrast to both Th1 and Th2 focused adjuvants that induce inflammation, Q11 nanofibers induce minimal inflammatory responses while still producing B-cell and T-cell responses (58), suggesting minimal direct interaction with TLRs or the adsorption of cytokines or other innate immune proteins that could trigger the inflammatory response. This suggests that Q11 adjuvanticity is independent of classical adjuvanting pathways which rely on inflammation for cellular recruitment, which may be one reason why antibody class switching was found here to be primarily driven by additional exogenous adjuvants and not by variations in the Q11 platform itself.

Although many anti-cytokine vaccines report total antibody titer for IgG, no publications to our knowledge report antibody subclass and isotype in the context of anti-cytokine immunization. This is in contrast to fields where vaccines are used to treat infectious disease. In these contexts, it is well-acknowledged that antibody subclass plays an active role in controlling viral infections (66–68). One posited role for this effect is the engagement of additional activating Fc Receptors in myeloid cell populations (56). In autoimmune conditions, where myeloid cell recruitment and complement activation are known to influence disease progression (69, 70), the same importance of antibody subclass is being actively explored with mAbs (71) but has yet to be shown *in vivo* with an anti-cytokine vaccination. Specifically for imiquimod-induced psoriasis, epidermal thickening via keratinocyte hyperproliferation is linked to both MyD88-dependent and MyD88-independent mechanisms and the recruitment of various myeloid populations (72). IgG2b, observed in the CpG vaccinated population, but not significantly in other adjuvanted conditions, also facilitates the recruitment of myeloid populations shown to be essential for IMQ psoriasis pathology (56).

Further, this study does not analyze the systemic effects of adjuvants such as CpG. CpG (ODN 1826) which acts as a TLR9 agonist (73), recruits many of the same cytokines that are essential for the IMQ-induced pathology (74). At sufficiently high doses systemic effects have been observed for as long as 8 days after administration (73, 75). Though this is significantly shorter than the 2.5 weeks between the final boost and beginning of IMQ application in this study it does not rule out at least some contribution of CpG systemic inflammation to the results reported here. Although the precise mechanism remains to be fully clarified, the replication of reduced therapeutic outcomes with CpG administration in both this and prior anti-cytokine therapies targeting TNF (38) suggests an important role for immune phenotype in the development of autologous antibody responses that will require further studies.

Additionally, the correlative nature of these conclusions highlights the limitations of our analysis of the IMQ-induced psoriasis model, which uses only epidermal thickening as the main metric for disease and does not examine different signaling pathways which may be responsible for the observed effects. The development and analysis of *in vitro* assays which are sensitive to both the specificity of induced-polyclonal antibodies for IL-17 protein in aqueous state and can account for effects induced by different Fc-receptor interactions will be necessary to characterize the mechanistic reason for the observed reductions in epidermal thickness. Assays not based on surface-bound antigen, which are subject to antigen denaturation or epitope occlusion, would also be able to more clearly measure the neutralizing characteristics of the antibodies raised against IL-17. Further investigation will also be required to determine if this is a phenomenon that is specific to the inhibition of inflammatory cytokines, or if the targeting of any endogenous protein will result in increased inflammation or pathology when the auto-antibody response has an IgG2b phenotype.

While tuning an anti-IL17 therapeutic in rodents has clear translational value in psoriasis, the role of this cytokine in other human disease appears to be less straightforward. Anti-IL-17 mAbs have not had the same success in rheumatoid arthritis, multiple sclerosis, or ankylosing spondylitis as has been seen in rodent models (3, 76, 77). While IL-17-dependent pathologies are seen in primary non-responders to other treatments such as anti-TNF therapy for rheumatoid arthritis (78), more commonly it is found that IL-17 synergistically promotes inflammation in several inflammatory diseases rather than acting as the primary driver of inflammation. This suggests that for future translation, a combination therapy or anti-cytokine immunization targeting multiple cytokines using a modular vaccine platform may provide an area of future exploration.

## Conclusion

We show here that peptide self-assemblies displaying B-cell epitopes from IL-17A and exogenous T-cell epitopes can raise IL-17A-specific antibodies and diminish symptoms of imiquimod-induced psoriasis in mice. The modularity of the system enabled a Design-of-Experiments approach where the influence of T-cell and B-cell epitope content on antibody titer and avidity could be investigated. Unadjuvanted nanofibers and those adjuvanted with alum had therapeutic efficacy in a mouse model of psoriasis, whereas formulations adjuvanted with CpG showed the opposite effect. Therapeutic efficacy was correlated with IgG1 but inversely correlated with IgG2b, illustrating the importance of tuning immune phenotype in active immunotherapies against cytokine targets.

## Data Availability Statement

The original contributions presented in the study are included in the article/Supplementary Material. Further inquiries can be directed to the corresponding author/s.

## Ethics Statement

The animal study was reviewed and approved by Duke Institutional Animal Care and Use Committee (IACUC).

## Author Contributions

LS designed experiments, conducted experiments, analyzed data, and wrote the manuscript. SK and KH designed experiments, conducted experiments, and analyzed data. JS designed experiments and conducted experiments. AM designed experiments and interpreted data. JC oversaw the project, designed experiments, analyzed data, and wrote the manuscript. All authors contributed to the article and approved the submitted version.

## Conflict of Interest

JC is listed as an inventor on patents associated with the technology described. AM is a consultant for Silab and has previously received project funding from Silab, but Silab had no decision on the content or the publishing of any of the manuscripts' contents. AM also consults for the Leo Foundation and The Triangle Community Foundation and receives honoraria. AM's spouse is employed by Precision Biosciences and holds stock and stock options. The remaining authors declare that the research was conducted in the absence of any commercial or financial relationships that could be construed as a potential conflict of interest.

## References

[1] Rodgers KR Chou RC. Therapeutic monoclonal antibodies and derivatives: historical perspectives and future directions. Biotechnol Adv.(2016) 34:1149–58. 10.1016/j.biotechadv.2016.07.004 27460206

[2] Rosenblum MD Remedios KA Abbas AK. Mechanisms of human autoimmunity. J Clin Investig. (2015) 125:2228–33. 10.1172/JCI78088 25893595 PMC4518692

[3] Siebert S Tsoukas A Robertson J Mcinnes I. Cytokines as therapeutic targets in rheumatoid arthritis and other inflammatory diseases. Pharmacol Rev. (2015) 67:280–309. 10.1124/pr.114.009639 25697599

[4] Ha H-L Wang H Pisitkun P Kim J-C Tassi I Tang W. IL-17 drives psoriatic inflammation via distinct, target cell-specific mechanisms. Proc Natl Acad Sci USA. (2014) 111:E3422–31. 10.1073/pnas.1400513111 25092341 PMC4143007

[5] Zagury D Burny A Gallo RC. Toward a new generation of vaccines: The anti-cytokine therapeutic vaccines. Proc Natl Acad Sci USA. (2001) 98:8024–9. 10.1073/pnas.141224798 11438746 PMC35461

[6] Assier E Bessis N Zagury JF Boissier MC. IL-1 vaccination is suitable for treating inflammatory diseases. Front Pharmacol. 8:6. 10.3389/fphar.2017.00006 28197099 PMC5281538

[7] Irani V Guy AJ Andrew D Beeson JG Ramsland PA Richards JS Molecular properties of human IgG subclasses and their implications for designing therapeutic monoclonal antibodies against infectious diseases. Mol Immunol. (2015) 67:171–82. 10.1016/j.molimm.2015.03.255 25900877

[8] Kang TH Jung ST. Boosting therapeutic potency of antibodies by taming Fc domain functions. Exp Mol Med. (2019) 51:138. 10.1038/s12276-019-0345-9 31735912 PMC6859160

[9] Bendtzen K Geborek P Svenson M Larsson L Kapetanovic MC Saxne T. Individualized monitoring of drug bioavailability and immunogenicity in rheumatoid arthritis patients treated with the tumor necrosis factor alpha inhibitor infliximab. Arthritis Rheum. (2006) 54:3782–9. 10.1002/art.22214 17133559

[10] Maneiro JR Salgado E Gomez-Reino JJ. Immunogenicity of monoclonal antibodies against tumor necrosis factor used in chronic immune-mediated Inflammatory conditions: systematic review and meta-analysis. JAMA Intern Med. (2013) 173:1416–28. 10.1001/jamainternmed.2013.7430 23797343

[11] St Clair EW Wagner CL Fasanmade AA Wang B Schaible T Kavanaugh A. The relationship of serum infliximab concentrations to clinical improvement in rheumatoid arthritis: results from ATTRACT, a multicenter, randomized, double-blind, placebo-controlled trial. Arthritis Rheum. (2002) 46:1451–9. 10.1002/art.10302 12115174

[12] Radstake TR Svenson M Eijsbouts AM Van Den Hoogen FH Enevold C Van Riel PL. Formation of antibodies against infliximab and adalimumab strongly correlates with functional drug levels and clinical responses in rheumatoid arthritis. Ann Rheum Dis. (2009) 68:1739–45. 10.1136/ard.2008.092833 19019895

[13] Bartelds GM Krieckaert CLM Nurmohamed MT Van Schouwenburg PA Lems WF Twisk JWR. Development of antidrug antibodies against adalimumab and association with disease activity and treatment failure during long-term follow-up. JAMA. (2011) 305:1460–8. 10.1001/jama.2011.406 21486979

[14] Ridker PM Tardif JC Amarenco P Duggan W Glynn RJ Jukema JW. Lipid-reduction variability and antidrug-antibody formation with bococizumab. N Engl J Med. (2017) 376:1517–26. 10.1056/NEJMoa1614062 28304227

[15] Delavallee L Duvallet E Semerano L Assier E Boissier MC. Anti-cytokine vaccination in autoimmune diseases. Swiss Med Week. (2010) 140:28–32. 10.4414/smw.2010.13108 21043003

[16] Mesin L Ersching J Victora GD. Germinal center B cell dynamics. Immunity. (2016) 45:471–82. 10.1016/j.immuni.2016.09.001 27653600 PMC5123673

[17] Pratt KP Anti-drug antibodies: emerging approaches to predict, reduce or reverse biotherapeutic immunogenicity. Antibodies. (2018) 7:19 10.3390/antib7020019 PMC6698869 31544871

[18] Wraith DC. Anti-cytokine vaccines and the immunotherapy of autoimmune diseases. Eur J Immunol. (2006) 36:2844–8. 10.1002/eji.200636760 17072913 PMC2615480

[19] Imamura CK. Therapeutic drug monitoring of monoclonal antibodies: Applicability based on their pharmacokinetic properties. Drug Metab Pharmacokin. (2019) 34:14–8. 10.1016/j.dmpk.2018.11.003 30606646

[20] Le Buanec H Delavallee L Bessis N Paturance S Bizzini B Gallo R. TNF alpha kinoid vaccination-induced neutralizing antibodies to TNF alpha protect mice from autologous TNF alpha-driven chronic and acute inflammation. Proc Natl Acad Sci USA. (2006) 103:19442–7. 10.1073/pnas.0604827103 17158801 PMC1748245

[21] Chackerian B Lowy DR Schiller JT. Conjugation of a self-antigen to papillomavirus-like particles allows for efficient induction of protective autoantibodies. J Clin Investig. (2001) 108:415–23. 10.1172/JCI11849 11489935 PMC209354

[22] Jennings GT Bachmann MF. Immunodrugs: therapeutic VLP-based vaccines for chronic diseases. Ann Rev Pharmacol Toxicol. (2009) 49:303–26. 10.1146/annurev-pharmtox-061008-103129 18851703

[23] Tissot AC Spohn G Jennings GT Shamshiev A Kurrer MO Windak R. A VLP-based vaccine against interleukin-1 alpha protects mice from atherosclerosis. Eur J Immunol. (2013) 43:716–22. 10.1002/eji.201242687 23254454

[24] Link A Bachmann MF. Immunodrugs: breaking B- but not T-cell tolerance with therapeutic anticytokine vaccines. Immunotherapy. (2010) 2:561–74. 10.2217/imt.10.30 20636009

[25] Uyttenhove C Van Snick J. Development of an anti-IL-17A auto-vaccine that prevents experimental auto-immune encephalomyelitis. Eur J Immunol. (2006) 36:2868–74. 10.1002/eji.200636662 17048276

[26] Foerster J Bachman M. Beyond passive immunization: toward a nanoparticle-based IL-17 vaccine as first in class of future immune treatments. Nanomedicine. (2015) 10:1361–9. 10.2217/nnm.14.215 25955128

[27] Zeltins A West J Zabel F El Turabi A Balke I Haas S. Incorporation of tetanus-epitope into virus-like particles achieves vaccine responses even in older recipients in models of psoriasis, Alzheimer's and cat allergy. NPJ Vaccines. (2017) 2:13. 10.1038/s41541-017-0030-8 29263885 PMC5653761

[28] Guan Q Weiss CR Qing G Ma Y Peng Z. An IL-17 peptide-based and virus-like particle vaccine enhances the bioactivity of IL-17 *in vitro* and *in vivo*. Immunotherapy. (2012) 4:1799–807. 10.2217/imt.12.129 23240747

[29] Lauwerys BR Hachulla E Spertini F Lazaro E Jorgensen C Mariette X. Down-regulation of interferon signature in systemic lupus erythematosus patients by active immunization with interferon α-kinoid. Arthritis Rheuma. (2013) 65:447–56. 10.1002/art.37785 23203821

[30] Durez P Vandepapeliere P Miranda P Toncheva A Berman A Kehler T. Therapeutic vaccination with TNF-kinoid in TNF antagonist-resistant rheumatoid arthritis: a phase II randomized, controlled clinical trial. PLoS ONE. (2014) 9:e113465. 10.1371/journal.pone.0113465 25517733 PMC4269456

[31] Cavelti-Weder C Timper K Seelig E Keller C Osranek M Lassing U. Development of an interleukin-1 beta vaccine in patients with type 2 diabetes. Mol Therapy. (2016) 24:1003–12. 10.1038/mt.2015.227 26686385 PMC4881764

[32] Ducreux J Houssiau FA Vandepapelière P Jorgensen C Lazaro E Spertini F Interferon α kinoid induces neutralizing anti-interferon α antibodies that decrease the expression of interferon-induced and B cell activation associated transcripts: analysis of extended follow-up data from the interferon α kinoid phase I/II study. Rheumatology. (2016) 55:1901–5. 10.1093/rheumatology/kew262 27354683 PMC5034220

[33] Rincon-Restrepo M Mayer A Hauert S Bonner DK Phelps EA Hubbell JA. Vaccine nanocarriers: coupling intracellular pathways and cellular biodistribution to control CD4 vs CD8 T cell responses. Biomaterials. (2017) 132:48–58. 10.1016/j.biomaterials.2017.03.047 28407494

[34] Rohner NA Thomas SN. Flexible macromolecule versus rigid particle retention in the injected skin and accumulation in draining lymph nodes are differentially influenced by hydrodynamic size. ACS Biomater Sci Eng. (2017) 3:153–9. 10.1021/acsbiomaterials.6b00438 29888321 PMC5990040

[35] Meyer RA Sunshine JC Perica K Kosmides AK Aje K Schneck JP. Biodegradable nanoellipsoidal artificial antigen presenting cells for antigen specific T-cell activation. Small. (2015) 11:1519–25. 10.1002/smll.201402369 25641795 PMC4529071

[36] Frey M Bobbala S Karabin N Scott E. Influences of nanocarrier morphology on therapeutic immunomodulation. Nanomedicine. (2018) 13:1795–811. 10.2217/nnm-2018-0052 30084296 PMC7270889

[37] Wen Y Waltman A Han HF Collier JH. Switching the immunogenicity of peptide assemblies using surface properties. Acs Nano. (2016) 10:9274–86. 10.1021/acsnano.6b03409 27680575 PMC5704984

[38] Mora-Solano C Wen Y Han HF Chen JJ Chong AS Miller ML. Active immunotherapy for TNF-mediated inflammation using self-assembled peptide nanofibers. Biomaterials. (2017) 149:1–11. 10.1016/j.biomaterials.2017.09.031 28982051 PMC5716349

[39] Rudra JS Tian YF Jung JP Collier JH. A self-assembling peptide acting as an immune adjuvant. Proc Natl Acad Sci USA. (2010) 107:622–7. 10.1073/pnas.0912124107 20080728 PMC2818904

[40] Rudra JS Mishra S Chong AS Mitchell RA Nardin EH Nussenzweig V. Self-assembled peptide nanofibers raising durable antibody responses against a malaria epitope. Biomaterials. (2012) 33:6476–84. 10.1016/j.biomaterials.2012.05.041 22695068 PMC3392361

[41] Rudra JS Sun T Bird KC Daniels MD Gasiorowski JZ Chong AS. Modulating Adaptive Immune Responses to Peptide Self-Assemblies. Acs Nano. (2012) 6:1557–64. 10.1021/nn204530r 22273009 PMC3289747

[42] Pompano RR Chen JJ Verbus EA Han HF Fridman A Mcneely T. Titrating T-cell epitopes within self-assembled vaccines optimizes CD4+helper T cell and antibody outputs. Adv Healthcare Mater. (2014) 3:1898–908. 10.1002/adhm.201400137 24923735 PMC4227912

[43] Jung JP Moyano JV Collier JH. Multifactorial optimization of endothelial cell growth using modular synthetic extracellular matrices. Integrative Biol. (2011) 3:185–96. 10.1039/c0ib00112k 21249249 PMC3401080

[44] Noack M Miossec P. Th17 and regulatory T cell balance in autoimmune and inflammatory diseases. Autoimmun Rev. (2014) 13:668–77. 10.1016/j.autrev.2013.12.004 24418308

[45] Blauvelt A Chiricozzi A. The immunologic role of IL-17 in psoriasis and psoriatic arthritis pathogenesis. Clin Rev Allergy Immunol. (2018) 55:379–90. 10.1007/s12016-018-8702-3 30109481 PMC6244934

[46] Von Stebut E Boehncke W-H Ghoreschi K Gori T Kaya Z Thaci D. IL-17A in psoriasis and beyond: cardiovascular and metabolic implications. Front Immunol. (2019) 10:3096. 10.3389/fimmu.2019.03096 32010143 PMC6974482

[47] Kolaskar AS Tongaonkar PC. A semi-empirical method for prediction of antigenic determinants on protein antigens. FEBS Lett. (1990) 276:172–4. 10.1016/0014-5793(90)80535-Q 1702393

[48] Wu YY Norberg PK Reap EA Congdon KL Fries CN Kelly SH. A supramolecular vaccine platform based on alpha-helical peptide nanofibers. Acs Biomater Sci Eng. (2017) 3:3128–32. 10.1021/acsbiomaterials.7b00561 30740520 PMC6364304

[49] Garner JP Weisker SM Dufour B Mench JA. Barbering (Fur and whisker trimming) by laboratory mice as a model of human trichotillomania and obsessive-compulsive spectrum disorders. Comp Med. (2004) 54:216–24. 15134369

[50] Alexander J Sidney J Southwood S Ruppert J Oseroff C Maewal A. Development of high potency universal Dr-restricted helper epitopes by modification of high-affinity Dr-blocking peptides. Immunity. (1994) 1:751–61. 10.1016/S1074-7613(94)80017-0 7895164

[51] Franke ED Hoffman SL Sacci JB Wang R Charoenvit Y Appella E. Pan DR binding sequence provides T-cell help for induction of protective antibodies against *Plasmodium yoelii* sporozoites. Vaccine. (1999) 17:1201–5. 10.1016/S0264-410X(98)00341-7 10195633

[52] Murphy KC Whitehead J Falahee PC Zhou D Simon SI Leach JK. Multifactorial experimental design to optimize the anti-inflammatory and proangiogenic potential of mesenchymal stem cell spheroids. Stem Cells. (2017) 35:1493–504. 10.1002/stem.2606 28276602 PMC5446296

[53] Kramer RM Archer MC Orr MT Dubois Cauwelaert N Beebe EA Huang P-WD. Development of a thermostable nanoemulsion adjuvanted vaccine against tuberculosis using a design-of-experiments approach. Int J Nanomed. (2018) 13:3689–711. 10.2147/IJN.S159839 29983563 PMC6028350

[54] Patel A Erb SM Strange L Shukla RS Kumru OS Smith L. Combined semi-empirical screening and design of experiments (DOE) approach to identify candidate formulations of a lyophilized live attenuated tetravalent viral vaccine candidate. Vaccine. (2018) 36:3169–79. 10.1016/j.vaccine.2017.04.086 28506515

[55] Morgan MT Bennett MT Drohat AC Kinetic analysis of the removal of halogenated uracil by human thymine DNA glycosylase. Effects of altering the CpG site context. Faseb J. (2007) 21:A291 10.1096/fasebj.21.5.A291-d

[56] Bruhns P. Properties of mouse and human IgG receptors and their contribution to disease models. Blood. (2012) 119:5640–9. 10.1182/blood-2012-01-380121 22535666

[57] Li W Joshi MD Singhania S Ramsey KH Murthy AK. Peptide vaccine: progress and challenges. Vaccines. (2014) 2:515–36. 10.3390/vaccines2030515 26344743 PMC4494216

[58] Chen JJ Pompano RR Santiago FW Maillat L Sciammas R Sun T. The use of self-adjuvanting nanofiber vaccines to elicit high-affinity B cell responses to peptide antigens without inflammation. Biomaterials. (2013) 34:8776–85. 10.1016/j.biomaterials.2013.07.063 23953841 PMC3814015

[59] Kelly SH Wu Y Varadhan AK Curvino EJ Chong AS Collier JH. Enabling sublingual peptide immunization with molecular self-assemblies. Biomaterials. (2020) 241:119903. 10.1016/j.biomaterials.2020.119903 32143059 PMC7171596

[60] Wu Y Kelly SH Sanchez-Perez L Sampson JH Collier JH. Comparative study of α-helical and β-sheet self-assembled peptide nanofiber vaccine platforms: influence of integrated T-cell epitopes. Biomater Sci. (2020) 8:3522–35. 10.1039/D0BM00521E 32452474 PMC7665831

[61] Sun T Han H Hudalla GA Wen Y Pompano RR Collier JH. Thermal stability of self-assembled peptide vaccine materials. Acta Biomater. (2016) 30:62–71. 10.1016/j.actbio.2015.11.019 26584836 PMC4821069

[62] Paul WE Zhu JF. How are T(H)2-type immune responses initiated and amplified? Nat Rev Immunol. (2010) 10:225–35. 10.1038/nri2735 20336151 PMC3496776

[63] Reed SG Orr MT Fox CB. Key roles of adjuvants in modern vaccines. Nat Med. (2013) 19:1597–608. 10.1038/nm.3409 24309663

[64] Smith DM Simon JK Baker JR. Applications of nanotechnology for immunology. Nat Rev Immunol. (2013) 13:592. 10.1038/nri3488 23883969 PMC7097370

[65] Bonam SR Partidos CD Halmuthur SKM Muller S. An overview of novel adjuvants designed for improving vaccine efficacy. Trends Pharmacol Sci. (2017) 38:771–93. 10.1016/j.tips.2017.06.002 28668223

[66] Huber VC Mckeon RM Brackin MN Miller LA Keating R Brown SA. Distinct contributions of vaccine-induced immunoglobulin G1 (IgG1) and IgG2a antibodies to protective immunity against influenza. Clin Vaccine Immunol. (2006) 13:981–90. 10.1128/CVI.00156-06 16960108 PMC1563571

[67] Mehlhop E Ansarah-Sobrinho C Johnson S Engle M Fremont DH Pierson TC. Complement protein C1q inhibits antibody-dependent enhancement of flavivirus infection in an IgG subclass-specific manner. Cell Host Microbe. (2007) 2:417–26. 10.1016/j.chom.2007.09.015 18078693 PMC2194657

[68] Su B Dispinseri S Iannone V Zhang T Wu H Carapito R. Update on Fc-mediated antibody functions against HIV-1 beyond neutralization. Front Immunol. (2019) 10:2968. 10.3389/fimmu.2019.02968 31921207 PMC6930241

[69] Fairweather D Cihakova D. Alternatively activated macrophages in infection and autoimmunity. J Autoimmun. (2009) 33:222–30. 10.1016/j.jaut.2009.09.012 19819674 PMC2783278

[70] Chen M Daha MR Kallenberg CGM The complement system in systemic autoimmune disease. J Autoimmun. (2010) 34:J276–86. 10.1016/j.jaut.2009.11.014 20005073

[71] Chan AC Carter PJ. Therapeutic antibodies for autoimmunity and inflammation. Nat Rev Immunol. (2010) 10:301–16. 10.1038/nri2761 20414204

[72] Costa S Marini O Bevilacqua D Defranco AL Hou BD Lonardi S. Role of MyD88 signaling in the imiquimod-induced mouse model of psoriasis: focus on innate myeloid cells. J Leukocyte Biol. (2017) 102:791–803. 10.1189/jlb.3MA0217-054RR 28642279 PMC6608051

[73] Liu H Moynihan KD Zheng Y Szeto GL Li AV Huang B. Structure-based programming of lymph-node targeting in molecular vaccines. Nature. (2014) 507:519–22. 10.1038/nature12978 24531764 PMC4069155

[74] Lai C-Y Su Y-W Lin K-I Hsu L-C Chuang T-H. Natural modulators of endosomal toll-like receptor-mediated psoriatic skin inflammation. J Immunol Res. (2017) 2017:7807313. 10.1155/2017/7807313 28894754 PMC5574364

[75] Von Beust BR Johansen P Smith KA Bot A Storni T Kündig TM. Improving the therapeutic index of CpG oligodeoxynucleotides by intralymphatic administration. Eur J Immunol. (2005) 35:1869–76. 10.1002/eji.200526124 15909311

[76] Schett G Elewaut D Mcinnes IB Dayer J-M Neurath MF. How cytokine networks fuel inflammation: toward a cytokine-based disease taxonomy. Nat Med. (2013) 19:822–4. 10.1038/nm.3260 23836224

[77] Mcinnes IB Buckley CD Isaacs JD. Cytokines in rheumatoid arthritis - shaping the immunological landscape. Nat Rev Rheumatol. (2016) 12:63–8. 10.1038/nrrheum.2015.171 26656659

[78] Alzabin S Abraham SM Taher TE Palfreeman A Hull D Mcnamee K. Incomplete response of inflammatory arthritis to TNF alpha blockade is associated with the Th17 pathway. Ann Rheumat Dis. (2012) 71:1741–8. 10.1136/annrheumdis-2011-201024 22550316

