# Supplementary material for: Multifactorial Design of a Supramolecular Peptide Anti-IL-17 Vaccine Toward the Treatment of Psoriasis
Source: Front Immunol. 2020 Aug 18;11:1855. doi: 10.3389/fimmu.2020.01855 (PMC7461889; doi:10.3389/fimmu.2020.01855)
Supplement: Supplementary file 1 [file Data_Sheet_1.PDF]

Supplemental Information

Table S1. Peptides Investigated

Peptide Name	Sequence	Molecular Weight (Da)
IL17.1-Q11	NH_2-HRNEDPDRYPSVIWE-SGSG-QQKFQFQFEQQ-NH_2	3668
IL17.2-Q11	NH_2-EAKDLFQ-SGSG-QQKFQFQFEQQ-NH_2	2605
biotin-IL17.1	Biotin-HRNEDPDRYPSVIWE-NH_2	2139
biotin-IL17.2	Biotin-EAKDLFQ-NH_2	1076
Q11	Ac-QQKFQFQFEQQ-NH_2	1527
PADRE-Q11	NH_2-aKXVAAWTLKAa-SGSG-QQKFQFQFEQQ-NH_2 (X = L-cyclohexylalanine, a = D-alanine)	3040

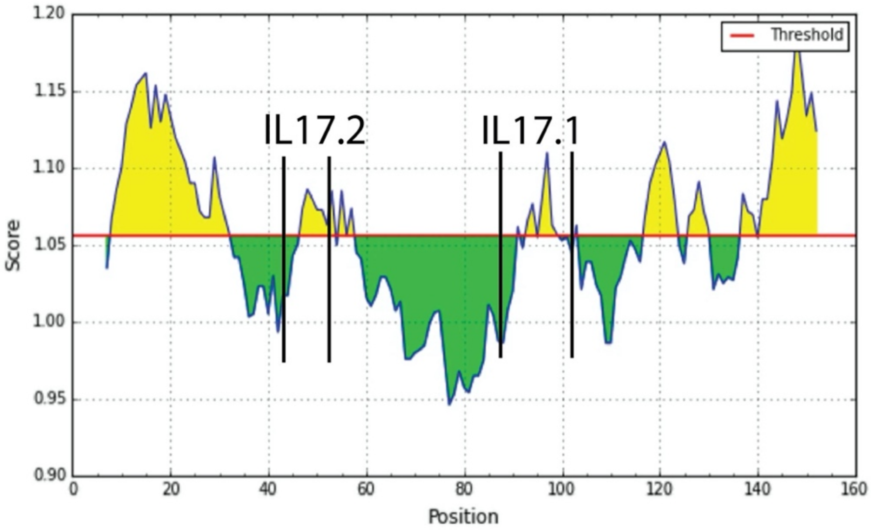

Figure S1. Kolaskar Tongaonkar Antigenicity predictions were used to detect peptide sequences with a high probability of acting as immunogenic B cell epitopes. Two epitopes (IL17.1 and IL17.2) were chosen from these predictions and by surface availability in homologous available crystal structures of human IL17A.

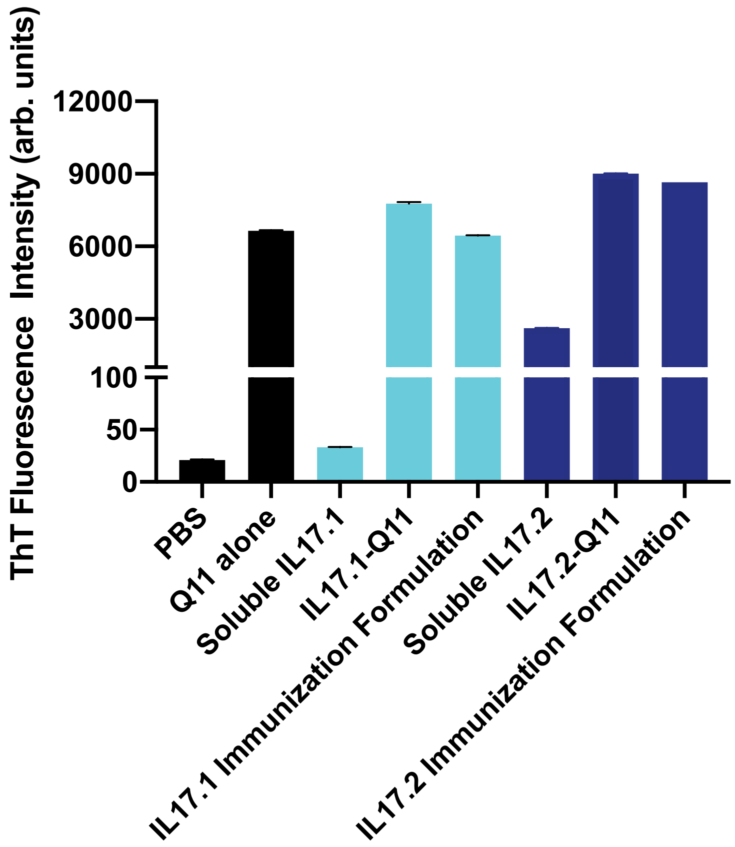

Figure S2. β-sheet nanofiber content was assessed using Thioflavin T (ThT). All groups were significantly different from all other groups (n =3) with the exception of PBS and IL17.1 peptide, which were not significantly different. Statistical significances were measured by One-way ANOVA and Tukey's Multiple Comparisons post-hoc analysis. Data are presented as Mean ± SD.

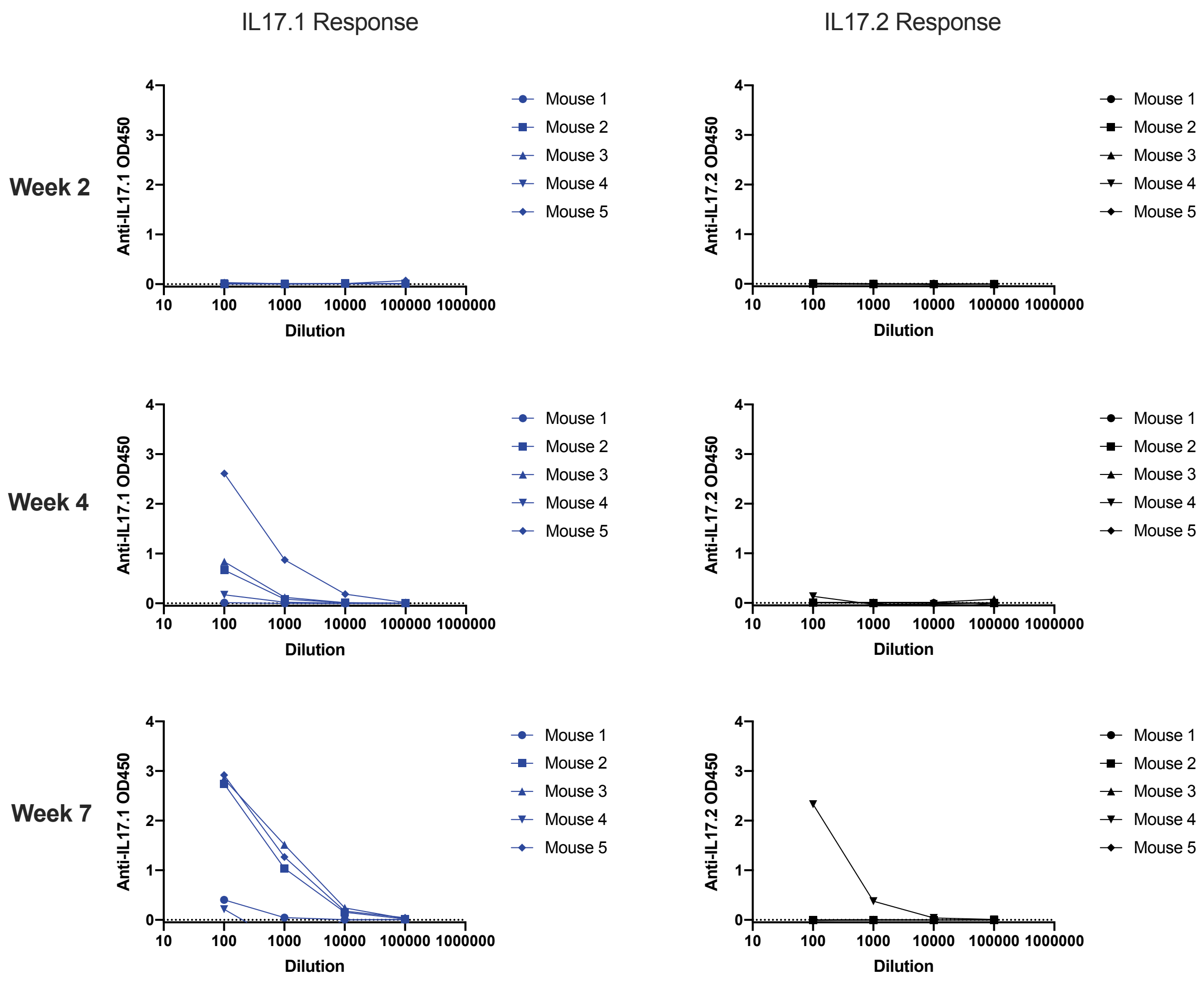

Figure S3. OD450 Values as a function of serum dilution for antibody titer responses in Figure 2A-B.

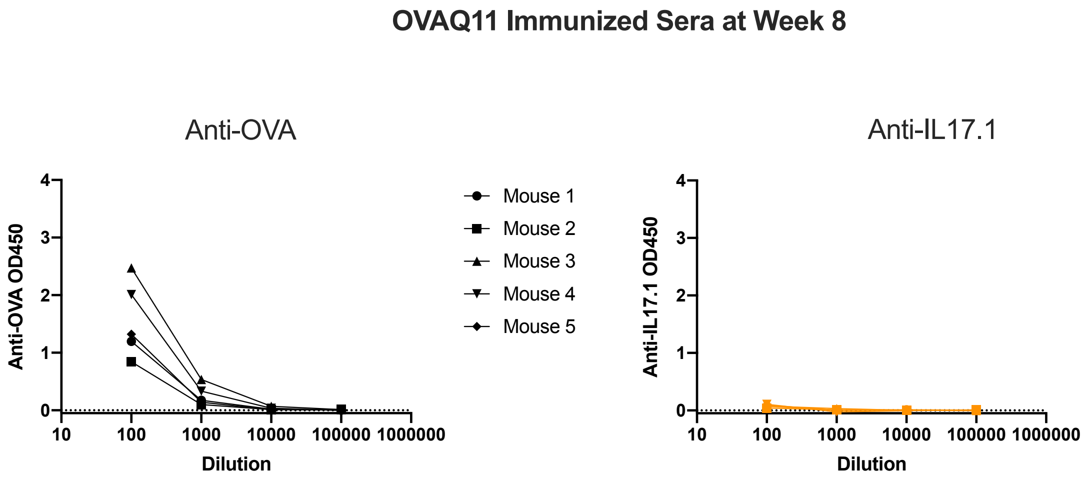

Figure S4. Anti-OVA peptide and anti-IL17.1 peptide responses from OVAQ11 immunized mice at week 8. Serum from mice with week 8 data previously published (Wu et al. *Biomaterials Science* 2020)

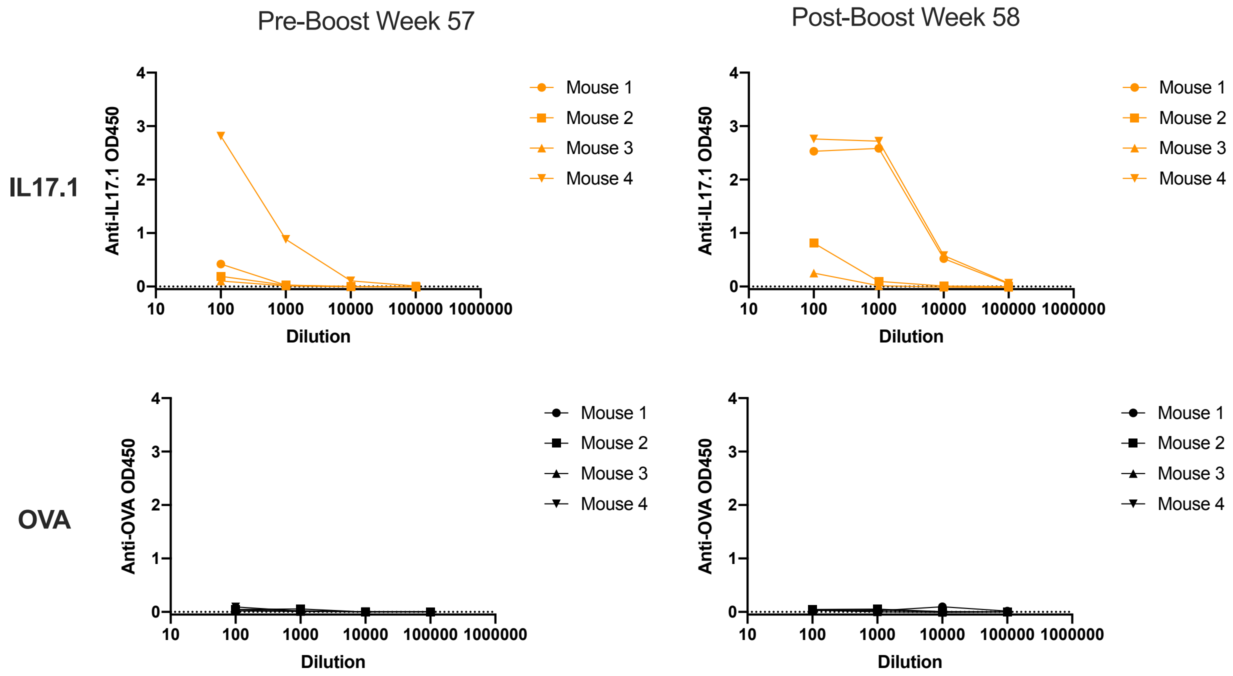

Figure S5 OD450 Values as a function of serum dilution for antibody titer responses in Figure Figure 3B.

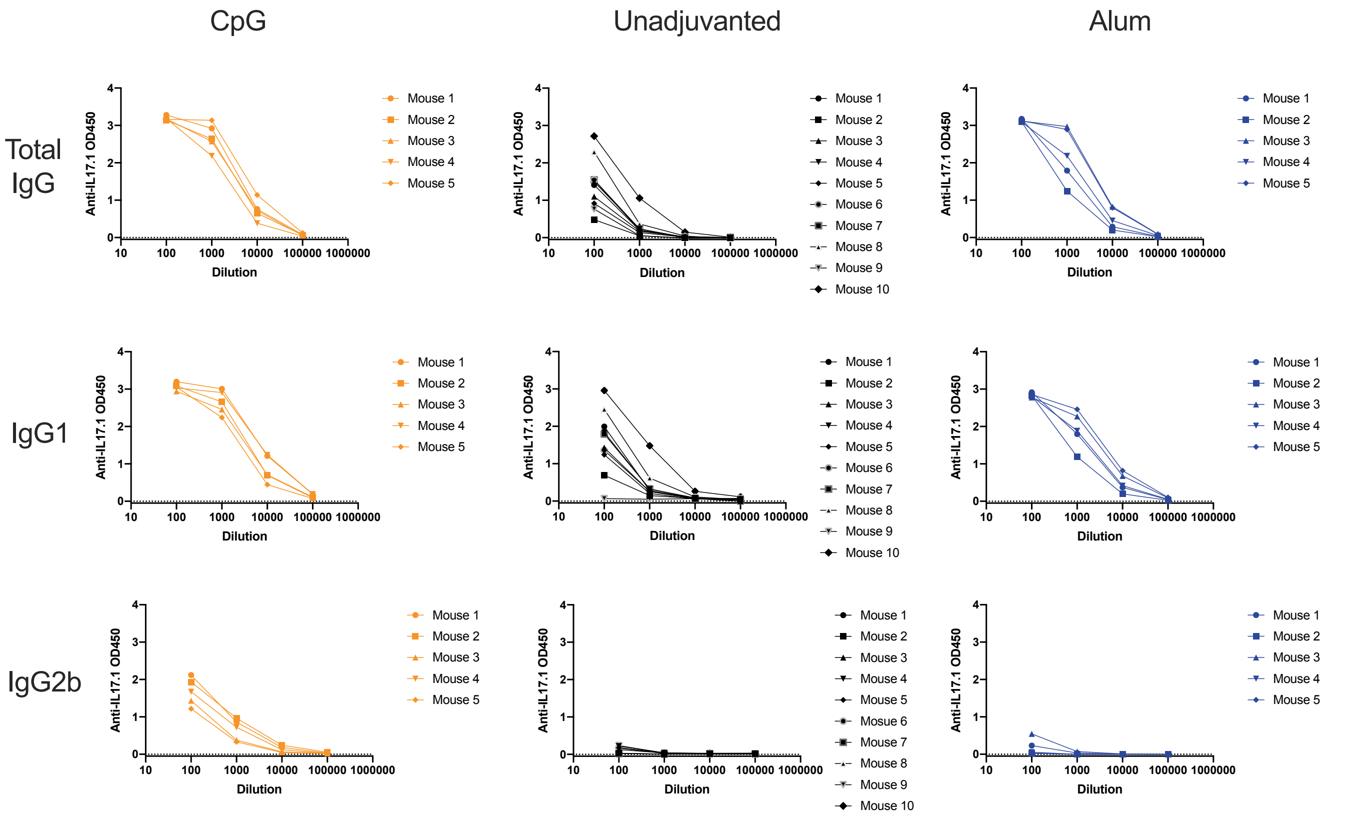

Figure S6. OD450 Values as a function of serum dilution for antibody titer responses in Figure Figure 5.

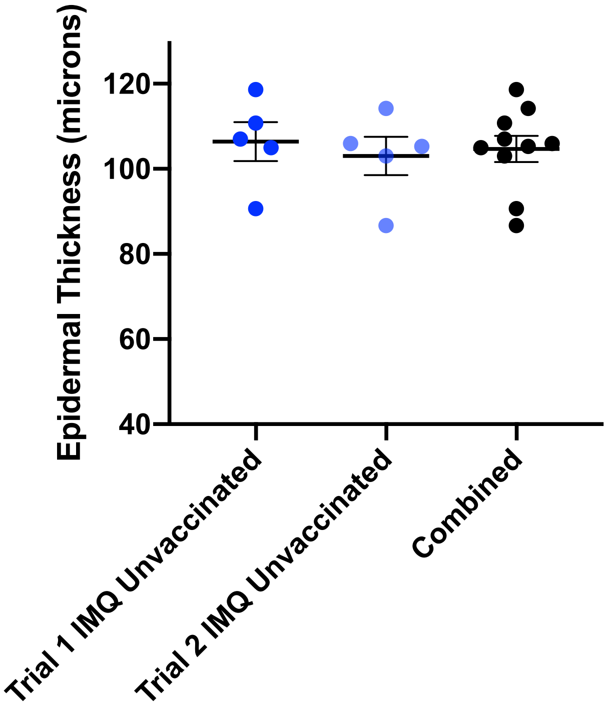

Figure S7. To justify combining two separately conducted experiments of IMQ-induced psoriasis, the positive control mice in both experiments (unvaccinated mice administered with IMQ) were compared between experiments for statistical differences. The average thickness (Mean +/- SD) in each experiment were 106.4 +/- 10.24 and 103.0 +/- 10.06, respectively. This indicated no significant difference between control groups by Student's t-test ($p = .6139$), so the experimental data were combined.

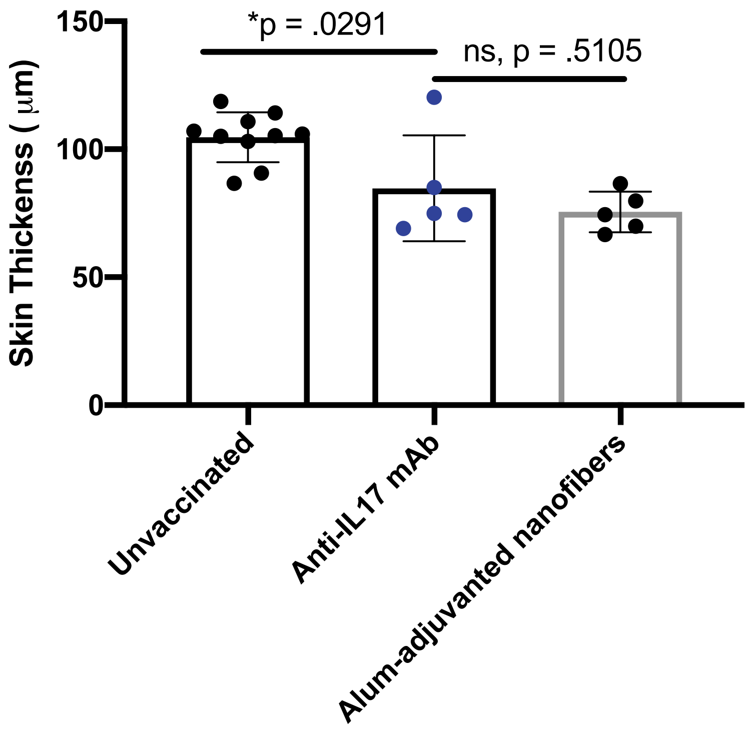

Figure S8. Anti-murine monoclonal antibody treatment significantly reduced epidermal thickening compared to unvaccinated controls. Unvaccinated mice that received anti-murine IL17 mAb three days before and on the same day as the application of imiquimod had significantly reduced epidermal thickening compared to unvaccinated control but no significant difference compared to alum-adjuvanted immunizations. Statistical significance was measured by One-way ANOVA and Tukey's Multiple Comparisons post-hoc analysis. * $p<0.05$ Data are presented as Mean ± SD.
